# Neuroserpin Is Strongly Expressed in the Developing and Adult Mouse Neocortex but Its Absence Does Not Perturb Cortical Lamination and Synaptic Proteome

**DOI:** 10.3389/fnana.2021.627896

**Published:** 2021-02-23

**Authors:** Dilara Kement, Rebecca Reumann, Katrin Schostak, Hannah Voß, Sara Douceau, Matthias Dottermusch, Michaela Schweizer, Hartmut Schlüter, Denis Vivien, Markus Glatzel, Giovanna Galliciotti

**Affiliations:** ^1^Institute of Neuropathology, University Medical Center Hamburg-Eppendorf, Hamburg, Germany; ^2^Institute of Clinical Chemistry and Laboratory Medicine, Mass Spectrometric Proteomics Group, University Medical Center Hamburg-Eppendorf, Hamburg, Germany; ^3^Physiopathology and Imaging of Neurological Disorders, Université Caen Normandie, INSERM U1237, Normandie Université, Caen, France; ^4^Department of Electron Microscopy, Center for Molecular Neurobiology, University Medical Center Hamburg-Eppendorf, Hamburg, Germany; ^5^Department of Clinical Research, Caen-Normandie University Hospital, Centre Hospitalier Universitaire, Caen, France

**Keywords:** neuroserpin, tissue-type plasminogen activator, nervous system development, cerebral cortex, synapse

## Abstract

Neuroserpin is a serine protease inhibitor that regulates the activity of tissue-type plasminogen activator (tPA) in the nervous system. Neuroserpin is strongly expressed during nervous system development as well as during adulthood, when it is predominantly found in regions eliciting synaptic plasticity. In the hippocampus, neuroserpin regulates developmental neurogenesis, synaptic maturation and in adult mice it modulates synaptic plasticity and controls cognitive and social behavior. High expression levels of neuroserpin in the neocortex starting from prenatal stage and persisting during adulthood suggest an important role for the serpin in the formation of this brain region and in the maintenance of cortical functions. In order to uncover neuroserpin function in the murine neocortex, in this work we performed a comprehensive investigation of its expression pattern during development and in the adulthood. Moreover, we assessed the role of neuroserpin in cortex formation by comparing cortical lamination and neuronal maturation between neuroserpin-deficient and control mice. Finally, we evaluated a possible regulatory role of neuroserpin at cortical synapses in neuroserpin-deficient mice. We observed that neuroserpin is expressed starting from the beginning of corticogenesis until adulthood throughout the neocortex in several classes of glutamatergic projection neurons and GABA-ergic interneurons. However, in the absence of neuroserpin we did not detect any alteration either in cortical layer formation, or in neuronal soma size and dendritic length. Furthermore, no significant quantitative changes were observed in the proteome of cortical synapses upon neuroserpin deficiency. We conclude that, although strongly expressed in the neocortex, absence of neuroserpin does not lead to gross developmental abnormalities, and does not perturb the composition of the cortical synaptic proteome.

## Introduction

Cognition, sensation, perception, voluntary movements are higher-order brain functions that rely on the ordered architecture of the cerebral cortex. The neocortex is organized into six histologically distinct layers containing two main classes of neurons: excitatory glutamatergic projection neurons and inhibitory GABA-ergic, mainly local circuit interneurons (Molyneaux et al., 2007). The first projection neurons appear at embryonic day (E) 10.5 in the mouse brain, during the following seven days projection neurons populating layers II–VI are generated in the ventricular zone and subventricular zone and migrate radially along radial glia into the cortical plate so that later born neurons pass earlier born neurons in the deeper layers V–VI to reach the more superficial layers II–IV in an “inside-out” fashion (Rakic, 1974; Molyneaux et al., 2007; Greig et al., 2013). GABA-ergic interneurons are generated from progenitors located in the ganglionic eminence during the same embryonic period as glutamatergic projection neurons and migrate tangentially into the neocortex (de Carlos et al., 1996; Tamamaki et al., 1997; Sultan et al., 2013). Once they reach their terminal destination, neurons undergo maturation by extending dendrites and establishing synaptic contacts with other cells, thereby forming neural circuits (Ohtaka-Maruyama and Okado, 2015). The coordinated generation and migration of cortical neurons is crucial for proper formation and functioning of the neocortex, as impairment of corticogenesis may lead to brain malformations or psychiatric disorders.

Neuroserpin is a serine protease inhibitor of the nervous system, regulating the activity of tPA in the brain (Galliciotti and Sonderegger, 2006). Dysregulated levels of neuroserpin have been observed in brain of patients affected by schizophrenia (Hakak et al., 2001; Vawter et al., 2004; Brennand et al., 2011; Wen et al., 2014). Neuroserpin expression is detected as early as E13 in the developing brain, first weakly and homogeneously throughout most central nervous system regions, then starting from E15 neuroserpin levels rise, especially in neocortex, hippocampus, cerebellar primordium, pons and medulla (Krueger et al., 1997). In the adult brain, neuroserpin expression is more restricted to areas where synaptic changes are associated with learning and memory. In the last years we uncovered an essential role of neuroserpin in the hippocampus. During developmental neurogenesis the serpin regulates neuronal precursor's proliferation and differentiation, and during the critical period it plays a role in synaptic maturation (Hermann et al., 2020). Moreover, in the adult brain, absence of neuroserpin is responsible for reduction of synapse density, deficits in synaptic plasticity, and alterations in hippocampal-dependent behavioral tasks (Reumann et al., 2017).

Recently, neuroserpin has been shown to reduce tPA-dependent Reelin cleavage in HEK293 cells (Krstic et al., 2012). Reelin is produced and secreted from Cajal-Retzius cells in the marginal zone of the developing cortex where it plays a fundamental role in neuronal migration and cortical lamination (Trotter et al., 2014; Jossin, 2020). Furthermore, it promotes growth of axons and dendrites as well as synaptogenesis. During adulthood, Reelin is expressed by GABA-ergic interneurons and controls synaptic plasticity. Several proteases cleave Reelin at different positions, producing proteolytic fragments that diffuse to distant brain regions, thereby regulating duration and range of Reelin signaling (Ranaivoson et al., 2016). For these reasons, regulation of Reelin cleavage by neuroserpin could represent a possible mechanism by which the serpin controls development and maintenance of the nervous system, including corticogenesis.

In this work, we present a detailed analysis of neuroserpin expression in the neocortex during brain formation. In particular, we performed immunohistochemical stainings to map cells expressing the neuroserpin protein (glutamatergic projection neurons, GABA-ergic interneurons, or glial cells) and investigate colocalization with its putative target, tPA. Moreover, to find out if neuroserpin influences cortical development by affecting Reelin proteolysis *in vivo*, we compared lamination, Reelin cleavage, neuronal morphology and perineuronal net composition between neuroserpin-deficient mice and control littermates. Finally, to gain insights into neuroserpin involvement at cortical synapses, we investigated the proteome of synaptosomes derived from the adult neocortex of mice lacking neuroserpin. We found a robust expression of neuroserpin in the somatosensory cortex starting from E13 until adulthood in several classes of projection neurons and interneurons, but no colocalization with GFAP-, Iba1- and Olig2-positive glial cell, indicating a specific neuronal source of neuroserpin. We localized tPA, the main target of neuroserpin, in the same cortical region expressing the serpin, however we never observed colocalization of both proteins within the same cells. Furthermore, we did not detect differences between neuroserpin-deficient mice and controls either in corticogenesis or in neuronal maturation and at the adult synapses. Expression of plasminogen activator inhibitor-1 (PAI-1), another serpin known to inhibit the proteolytic activity of tPA and possibly compensating for neuroserpin absence, was not altered in neuroserpin-deficient mice. Taken together, these results suggest that, despite its strong presence in the neocortex, neuroserpin is not indispensable for normal formation of this brain region.

## Materials and Methods

### Animals

Neuroserpin knockout mice (Ns-/-) have been described before (Madani et al., 2003), they were backcrossed to a C57BL/6J background for at least ten generations and wild-type (wt) littermates were used as controls. For the Golgi-Cox staining, C57BL/6J mice were used as control. Animals were generated in the animal facility of the University Medical Center Hamburg Eppendorf, Germany, and maintained in groups of 2–4 littermates under standard housing conditions with food and water *ad libitum*. Mice of both sexes were used (unless stated otherwise). Animal procedures were performed in accordance with the ARRIVE guidelines, the institutional guidelines from the animal facility of the University Medical Center Hamburg Eppendorf, Germany, and were registered at the local animal care agency (Behörde für Lebensmittelsicherheit und Veterinärwesen, registration number ORG-739). For neuroserpin-tPA colocalization study, pregnant C57BL/6J mice were in utero electroporated at E14 with pPlat-GFP and pCAG-RFP plasmids at the animal facility of the University of Caen, France, as previously published (Pasquet et al., 2019). The pCAG-RFP plasmid was purchased from Addgene. The pPlat-GFP plasmid (1.2-kb sequence of the human Plat promoter, spanning from −1035 to +207 base pairs from the transcription start codon) was purchased from GeneCopoeia (pPlat-GFP; Catalog No.: HPRM12655-PF02). Both constructs were amplified in Escherichia coli JM109 cells and purified by a Nucleobond endotoxin-free plasmid DNA PC 2000 kit (Macherey-Nagel) according to the manufacturer's instructions. Mouse protocols were performed in accordance with the French (Decree 87/848) and the European Communities Council (Directive 86/609) guidelines. The project was approved by the ethical committee CENOMEXA (under the identification number #10638). Information about animal used in this study can be found in Supplementary Table 1.

### Immunohistochemical Analysis of Brain Tissue

Mice were sacrificed at different time points (E13, E16, P0, 1, 3, and 13 weeks of age, three animals per group), for stages E13 and E16 the whole head was used, while for P0, 1, 3, and 13 weeks of age the brain was dissected before fixation. Tissues were post-fixed in 4% formaldehyde, cryoprotected in 30% sucrose and frozen in tissue freezing medium. For Nissl staining, 10 μm cryosections were processed according to standard protocols. For immunohistochemical staining, 10 μm cryosections were boiled for 10 min in 10 mM citrate buffer (10 mM sodium citrate, 0.05% Tween-20, pH 6.0) for antigen retrieval, permeabilized in PBS-0,2 % Triton X-100 and blocked in PBS-0,4% Triton X-100-5% BSA. Sections were incubated overnight at 4°C with affinity-purified anti-neuroserpin goat polyclonal antibody (1:50) (Galliciotti et al., 2007), primary antibody against Reelin (1:600, Merck Millipore MAB5364), Cux1 (1:300, Proteintech 11733-1-AP), Ctip2 (1:600, abcam ab18465), Tbr1 (1:1000, abcam ab31940), Parvalbumin (1:1,000, Sigma P3088), Somatostatin (1:50, Santa Cruz Biotechnology sc-74556), GFAP (1:500, Merck Millipore MAB360), Iba1 (1:500, abcam ab5076), Olig2 (1:200, Merck Millipore AB9610), aggrecan (1:50, Merck Millipore AB1031), MAP2 (1:100, Sigma M3696) or GFP (1:500, Clontech 632380) in blocking buffer containing 0,2 % Triton X-100. Anti-rabbit, anti-mouse, anti-rat or anti-goat conjugated to Alexa Fluor 555, Alexa Fluor 488 or Alexa Fluor 647 (1:500, Thermo Fisher) were used as secondary antibodies. Sections were covered (Fluoromount-G with DAPI, SouthernBiotech), dried and pictures were taken using a Leica TCS SP5 or SP8 confocal microscope, the Plan-APOCHROMAT 40x or 63x oil-immersion lens and 1,024 × 1,024 pixels for frame. For quantification of cortical immmunofluorescence signal, digital image analysis of anatomically matched control and mutant mice cortices was performed using ImageJ/Fiji software (Schindelin et al., 2012). Positive fluorescence signal was detected by consistent automatic global thresholding. Improper pixels corresponding to background noise were removed by median filtering. Cortical images were subsequently divided into 10 equally sized bins, in each of which the percentage of positive signal per area was measured. The quantification described was applied consistently to all images. Three animals per condition were analyzed.

### Golgi-Cox Staining

Brains (three animals per group) were collected from mice at 3 weeks of age, quickly rinsed with double-distilled water and stained using the FD Rapid GolgiStain Kit (FD NeuroTechnologies) following the manufacturer's instructions. Briefly, tissues were immersed in impregnation solution (A+B) at room temperature (RT) in the dark. After overnight incubation the solution was replaced, and brains further incubated for 1 week. Afterwards, tissues were transferred into solution C and stored 24 h at 4°C in the dark. Solution C was refreshed, and incubation prolonged for another 24 h. Brains were then embedded in 4% low melting point agarose and 100 μm sections were cut with a vibratome. Slices were rinsed with distilled water, incubated 10 min in staining solution (D + E) and after a last washing step with distilled water mounted on a slide with Mowiol and stored at 4°C in the dark. Pictures were taken using a Zeiss AxioVision light microscope. Soma size and dendrite length were measured using Stereo Investigator (MBF Bioscience). Length of dendrites was measured under the microscope at 20 × -magnification starting at the base of the soma and along all protrusions until the tip. Thirty cells per mouse from layers IV–VI were analyzed, average of soma size and average of all dendrites per animal were calculated (wt: 386, 406, and 439 dendrites; Ns-/-: 322, 384, and 418 dendrites per animal were quantified).

### Electron Microscopy

Brains were collected from Ns-/- mice and wt littermates (three animals per group, all males, 60 weeks of age). Sagittal vibratome sections of the primary somatosensory cortex were collected and prepared for electron microscopy as described (Koster et al., 2016). Ultrathin sections (60 nm) were examined in an EM902 (Zeiss). Pictures (15/animal) were taken from cortical layer IV with a MegaViewIII digital camera (A. Tröndle).

### Synaptosome Isolation

Brains were collected from Ns-/- mice and wt littermates (four animals per group, all males, 60 weeks of age), the neocortex was dissected and directly processed. Synaptosomes were prepared following the protocol published in (Phelan and Gordon-Weeks, 1997) with some modifications: Tissues were homogenized in solution A containing protease inhibitor cocktail (complete, Mini, EDTA-free, Roche) using a dounce homogenizer. After the isolation, synaptosomes were recovered from the sucrose gradient, diluted in four volumes of solution A-protease inhibitor cocktail and pelleted by centrifugation for 30 min at 26'000 × *g*, 4°C. The synaptosome pellet was snap-frozen and stored at −80°C. After thawing, samples were centrifuged for 30 min at 20'000 × *g*, 4°C and the pellet was subjected to mass spectrometric analysis.

### Tryptic Digestion for Mass Spectrometric Analysis

Protein pellets from cortical synaptosomes were lysed in triethylammonium hydrogen carbonate buffer (TEAB, Thermo Fisher) with 1% w/w sodium deoxycholate (SDC, Sigma Aldrich), boiled at 95°C for 5 min and sonicated five times at 30% using a probe sonicator to destroy DNA/RNA. 30 μg protein were used for tryptic digestion. Disulfide bridges were reduced in the presence of 10 mM dithiothreitol (DTT, Sigma Aldrich) at 60°C for 30 min and alkylated with 20 mM iodoacetamide (IAA, Sigma Aldrich) for 30 min in the dark at 37°C. Trypsin (sequencing grade, Promega) was added at a 1:100 ratio (enzyme to protein) and digestion was performed overnight at 37°C. The reaction was quenched and SDC was precipitated using 1% formic acid (FA, Fluka). Samples were centrifuged for 5 min at 14,000 × *g*, the supernatant was transferred into a new tube and lyophilized using a SpeedVac^TM^ vacuum concentrator.

### Mass Spectrometric Measurements and Data Analysis

The mass spectrometry proteomics data have been deposited to the ProteomeXchange Consortium *via* the PRIDE (https://www.ebi.ac.uk/pride/) partner repository with the dataset identifier PXD022371.

Prior to mass spectrometric analyses, peptides were resuspended in 0.1% FA to a final concentration of 1 μg/μl. LC-MS/MS measurements were performed on a quadrupole-iontrap-orbitrap mass spectrometer (Orbitrap Fusion, Thermo Fisher) coupled to a nano-UPLC (Dionex Ultimate 3000 UPLC system, Thermo Fisher). One micro gram of tryptic peptides were injected into the chromatographic system *via* an autosampler, purified and desalted using a reversed phase trapping column (Acclaim PepMap 100 C18 trap; 100 μm × 2 cm, 100 Å pore size, 5 μm particle size; Thermo Fisher) and transferred to a reversed phase column for chromatographic separation (Acclaim PepMap 100 C18; 75 μm × 50 cm, 100 Å pore size, 2 μm particle size, Thermo Fisher). Trapping was done for 5 min at a flow rate of 15 μl/min with 99% solvent A (0.1% FA) and 1% solvent B (0.1% FA in ACN). Separation and elution of peptides were achieved by a linear gradient from 1 to 30% solvent B in 70 min at a flow rate of 3 μl/min. Eluted peptides were ionized using a nano-electrospray ionization source (nano-ESI) with a spray voltage of 1,800, transferred into the mass spectrometer and analyzed in data dependent acquisition (DDA) mode. For each MS1 scan, ions were accumulated for a maximum of 120 ms or until a charge density of 2 × 105 ions (AGC Target) was reached.

Fourier-transformation-based mass analysis of the data from the orbitrap mass analyser was performed covering a mass range of 400–1,300 m/z with a resolution of 120,000 at m/z = 200. Peptides with charge states between 2+ and 5+ above an intensity threshold of 1,000 were isolated within a 1.6 m/z isolation window in Top speed mode for 3 s from each precursor scan and fragmented with a normalized collision energy of 30% using higher energy collisional dissociation (HCD). MS2 scanning was performed using an orbitrap mass analyser, covering a mass range of 380–1,500 m/z with a orbitrap resolution of 15'000 at m/z = 200 and accumulated for 60 ms or to an AGC target of 1 × 105. Dynamic exclusion of fragmented peptides was applied for 15 s after precursor selection.

Raw data obtained from LC-MS/MS measurements were processed with MaxQuant version 1.6.2.10 (Max Plank Institute for Biochemistry, Version 1.6.2.10) using the integrated Andromeda algorithm. For protein identification, measured MS2 spectra were searched against theoretical fragment-spectra of tryptic peptides, generated from a reviewed murine Swissprot FASTA database obtained in February 2020, containing 17,015 entries. All samples were handled as individual experiments.

The Carbamethylation of cysteine residues was set as a fixed modification. Methionine oxidation, protein N-terminal acetylation, removal of the initiator methionine at the protein N-terminus and the conversion of glutamine to pyroglutamate were set as variable modifications. Peptides with a minimum length of six amino acids and a maximum mass of 6,000 Da were identified with a mass tolerance of 10 ppm. Only peptides with maximum of two missed trypsin cleavage sites were considered. For peptide identification, matching between runs was included, using a match time window of 0.7 min and an alignment time window of 20 min between individual runs. The error tolerance was set to 20 ppm for the first precursor search and to 4.5 ppm for the following main search. Fragment spectra were matched with 20 ppm error tolerance. A false discovery rate (FDR) value threshold <0.01, using a reverted decoy peptide databases approach was set for peptide identification. Label free quantification was performed with an LFQ minimum ratio count of 1. For quantification, all identified razor and unique peptides were considered. The label minimum ratio count was set to 1.

### Protein Extraction

Tissues (whole brain for stages E13 and E16, neocortex for P0, 1, 3, and 13 weeks of age, 3 animals per group) were homogenized in 10 volumes of 20 mM Tris–HCl, pH 7.5, containing 150 mM NaCl, protease inhibitor cocktail (complete, Mini, EDTA-free, Roche) and PhosSTOP phosphatase inhibitor cocktail (Roche) using a dounce homogenizer (Reumann et al., 2017). Proteins were solubilized by the addition of Triton X-100 to a final concentration of 1%. Extracts were cleared from insoluble material by centrifugation for 30 min at 20'000 × *g*, 4°C. For analysis of perineuronal net (PNN), extracts were digested with 0.05 U Chondroitinase ABC (Sigma) for 2 h at 37°C (Hermann et al., 2020). Protein concentration was determined with Quick Start Bradford 1 × Dye Reagent (BioRad Laboratories) as described by the manufacturer.

### Western Blotting and Densitometry

Mouse cortical extracts (80 μg total protein) were electrophoretically separated on 8% (for Reelin, ADAMTS-4 and aggrecan) or 10% (for PAI-1) SDS-PAGE under reducing conditions. Proteins were transferred to nitrocellulose membranes (Bio-Rad Laboratories) and membranes were blocked for 1 h at RT with Roti®-ImmunoBlock (Carl Roth) in Tris-buffered saline. Primary antibodies [anti-Reelin, 1:1,000, MAB5364 Merck Millipore; anti-ADAMTS-4, 1:500, abcam ab185722; anti-PAI-1, 1:1,000, abcam ab222754; anti-aggrecan, 1:1,000, AB1031 Merck Millipore; anti-beta-actin clone C4, 1:4,000, MAB1501 Merck Millipore; affinity-purified anti-neuroserpin goat polyclonal antibody, 0,5 μg/ml, (Galliciotti et al., 2007)] were incubated overnight at 4°C in Tris-buffered saline containing 0.05% Tween-20 and Roti®-ImmunoBlock. Secondary antibodies conjugated with IRDye® 800CW or IRDye® 680RD (1:10'000) (LI-COR Biosciences) were incubated in the same buffer for 1 h at RT. Membranes were scanned using an Odyssey® Infrared Imaging System (LI-COR Biosciences). Densitometric quantification was performed with LI-COR® Odyssey Software, version 2.0 (LI-COR Biosciences) and local background subtraction. Band intensity was normalized to beta-actin expression, and values for the wt group were arbitrarily set to 1.

### Statistical Analysis

For quantification of Reelin, ADAMTS-4, PAI-1, and aggrecan expression and cleavage, soma size and dendrite length, statistical comparison among groups was determined using two-tailed Student's *t*-test (wt vs. Ns-/-). For statistical analysis of quantified cortical immmunofluorescence, signal detection fractions in matching bins were compared between control and mutant mice using two-tailed Student's *t*-test. *p* values were adjusted for multiple comparisons using the Holm-Sidak-method. Means +/– SD are reported, statistical significance was set at ^*^p ≤ 0.05, ^**^p ≤ 0.01 and ^***^p ≤ 0.001. The following statistical softwares were used: GraphPad Prism version 5.0 (GraphPad Software) and Excel (Microsoft-Office). For mass spectrometric analysis, the ProteinGroups.txt result file from MaxQuant was loaded into Perseus software (Max Plank Institute for Biochemistry, Version 1.5.8.5). The quantitative LFQ Intensity values for protein groups were used as main columns. The relative protein abundance for each protein was transformed into log2 values and normalized by subtraction of the median for each column. Protein lists were filtered for proteins, quantifiable in at least three samples of each group of wt and Ns-/-. Euclidian distance based unsupervised clustering as well as principle component analysis was performed to estimate the distinguishability of compared groups. Student's *t*-test was performed to identify statistically differential abundant proteins between wt and neuroserpin-deficient mice (*p*-value < 0.05). Only proteins exceeding a cutoff of 1.5-fold change difference between the queried groups were classified as differences of biological origin and considered in further analysis.

## Results

### Immunohistochemical Analysis of Neuroserpin Expression During Development of the Neocortex

**Figure 1:**
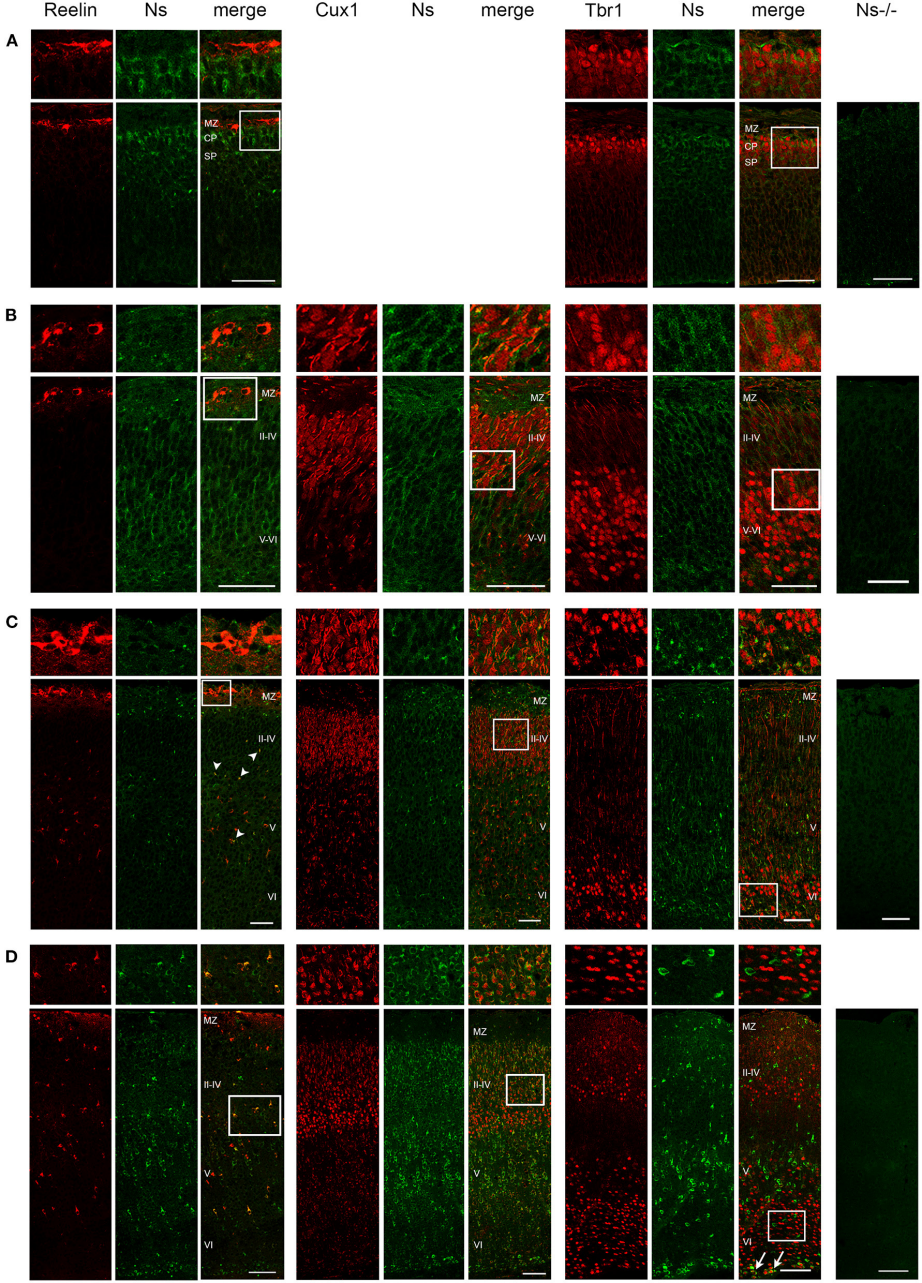
Neuroserpin expression during cortical development. Representative immunohistochemical staining of the somatosensory cortex with an antibody directed against neuroserpin (Ns) and markers of cortical layer I (Reelin), II–IV (Cux1) and V–VI (Tbr1) at E13 **(A)**, E16 **(B)**, P0 **(C)** and at 1 week of age **(D)**. At E13, upper cortical layers with Cux1-positive neurons have not yet been generated. Neuroserpin immunoreactivity is homogeneously observed throughout the developing cortical plate, whereas the Reelin-positive marginal zone is devoid of neuroserpin. In the postnatal period, neuroserpin-immunopositive cells are still distributed throughout cortical layer II–VI, around many Cux1-positive nuclei, whereas most Tbr1-positive neurons in the deeper layers do not express neuroserpin (arrows in **D**). Moreover, colocalization with Reelin-positive interneurons is observed starting from P0 (arrowheads in **C**). Staining of a cortical section from a Ns-/- mouse demonstrates specificity of the neuroserpin immunohistochemistry. White boxes represent the area shown at higher magnification in the top row. Scale bars: 50 μm in **(A**–**C)**, 100 μm in **(D)**. *n* = 3. MZ, marginal zone; CP, cortical plate; SP, subplate.

**Figure 2:**
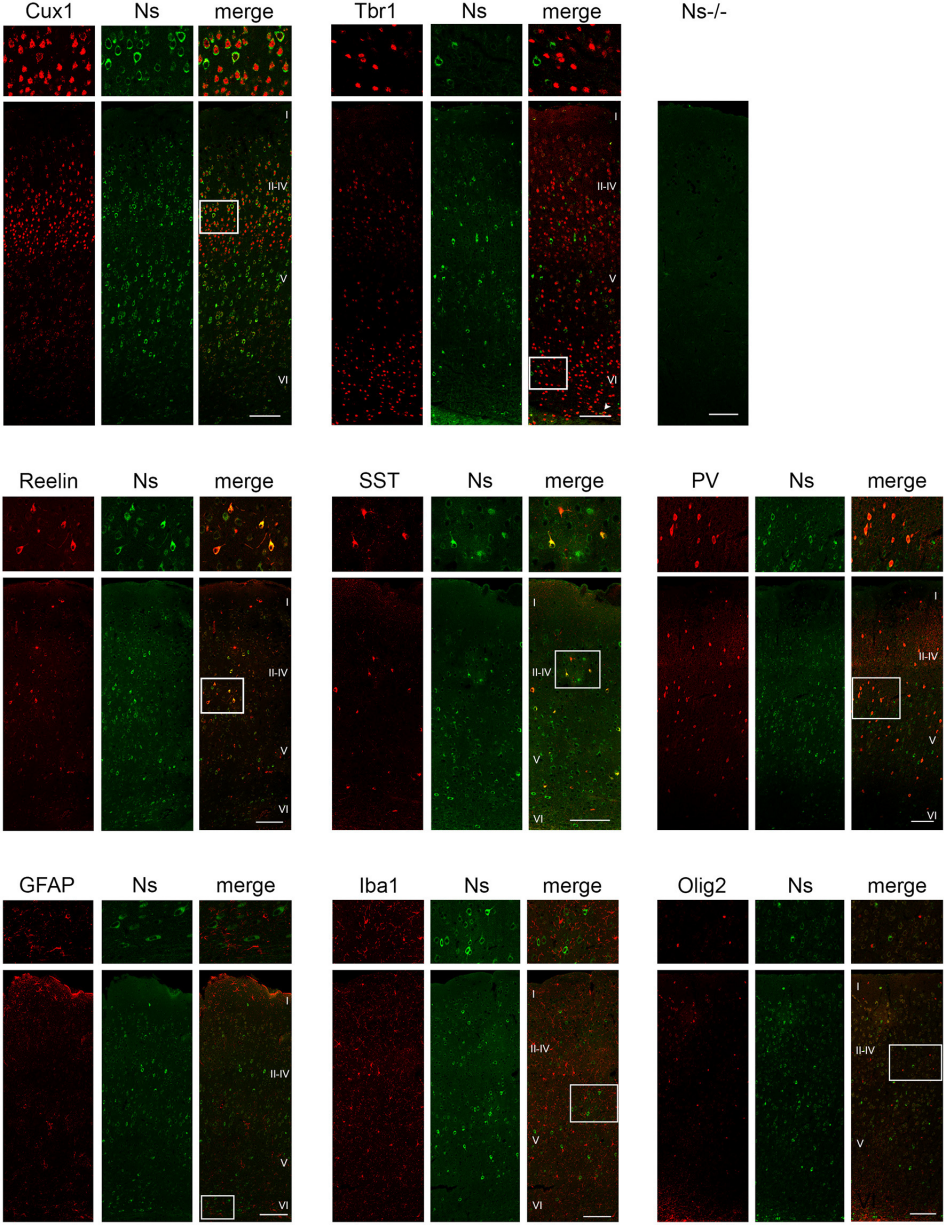
Neuroserpin expression in the adult somatosensory cortex. Neuroserpin (Ns) immunoreactivity is often observed in Cux1-positive, but barely in Tbr1-positive projection neurons. Moreover, neuroserpin is also expressed by Reelin-, SST-, and PV-positive interneurons throughout the cortex. GFAP-positive astrocytes, Iba1-positive microglia as well as Olig2-positive oligodendrocytes are devoid of neuroserpin staining. White boxes represent the area shown at higher magnification in the top row. Scale bars: 100 μm, *n* = 3.

We analyzed the distribution of neuroserpin expression in the somatosensory cortex during development and in the adulthood. We found neuroserpin immunoreactivity as early as E13 (Figure 1A). At this stage, Reelin-positive Cajal-Retzius cells were present in the marginal zone, however, they did not express neuroserpin, as no colocalization was observed between the two proteins. Instead, neuroserpin expression was clearly visible in the developing cortical plate at E13, where it was found around the nuclei of Tbr1-positive projection neurons. Three days later, at E16, the cortical plate has expanded and neuroserpin was expressed homogeneously at low levels throughout it (Figure 1B). Neuroserpin immunoreactivity was present around Cux1-positive pyramidal neuron nuclei of the newly formed layers II–IV and it persisted in the Tbr1-positive deep-layer neurons. Again, no colocalization could be observed between neuroserpin and Reelin in neurons of the marginal zone. At birth [postnatal day (P) 0] (Figure 1C) and in young 1 week-old animals (Figure 1D), when lamination of the neocortex is completed, neuroserpin was still localized throughout all layers of the cerebral cortex. However, in contrast to prenatal stages E13 and E16, at early postnatal stages during maturation of the neocortex neuroserpin immunoreactivity was predominantly present at high level in distinct cells. Reelin-positive Cajal-Retzius cells were still devoid of neuroserpin immunoreactivity, however Reelin-positive interneurons were now present in all cortical layers, and some showed immunoreactivity for neuroserpin. Moreover, neuroserpin immunostaining was again found in Cux1-positive pyramidal neurons of the upper layers. In deep cortical layers, although neuroserpin staining was visible, neurons expressing both Tbr1 and the serpin could hardly be found at 1 week of age, only few cells at the bottom of layer VI were double-positive for both proteins. Next, we studied neuroserpin localization in adult animals at 13 weeks of age (Figure 2). Neuroserpin expression was strongly detected throughout the cortex. Again, presence of neuroserpin was rather concentrated at high level in distinct cells, with the remaining cortical cells bearing low to undetectable levels of the serpin. In order to determine which cells express neuroserpin in the adult cortex, we performed colocalization with markers of projection neurons, interneurons and glial cells. Although in the superficial layers Cux1-positive neurons expressing neuroserpin were frequently detected, neurons expressing both Tbr1 and neuroserpin were rarely found in the deep cortical layers, in a pattern similar to the one observed in 1 week-old mice. Furthermore, neuroserpin immunoreactivity was observed in several Reelin-, somatostatin (SST)-, and parvalbumin (PV)-positive interneurons throughout the neocortex (Figure 2). Finally, neither glial fibrillary acidic protein (GFAP)-positive astrocytes, nor Iba1-positive microglia, nor Olig2-positive oligodendrocytes expressed neuroserpin, suggesting that neuroserpin expression is restricted to neuronal cells (Figure 2). Lastly, we investigated colocalization of neuroserpin with its target protease, tPA. Since neuroserpin expression is at most during the first postnatal week (Krueger et al., 1997), we chose the P0 time point for this analysis (Supplementary Figure 1). We examined the somatosensory cortex of mice previously in utero electroporated at E14 with a plasmid encoding for green fluorescence protein (GFP) under the control of the tPA promoter (Lenoir et al., 2019), resulting in a strong green fluorescence in tPA-expressing cells. We observed expression of both neuroserpin and tPA-GFP throughout all layers of the cerebral cortex. However, we never detected cells positive for both proteins. Thus, we concluded that neuroserpin and its target protease tPA are present in the same cortical region but are produced by different cells.

### Absence of Neuroserpin Does Not Alter Lamination of the Neocortex

**Figure 3:**
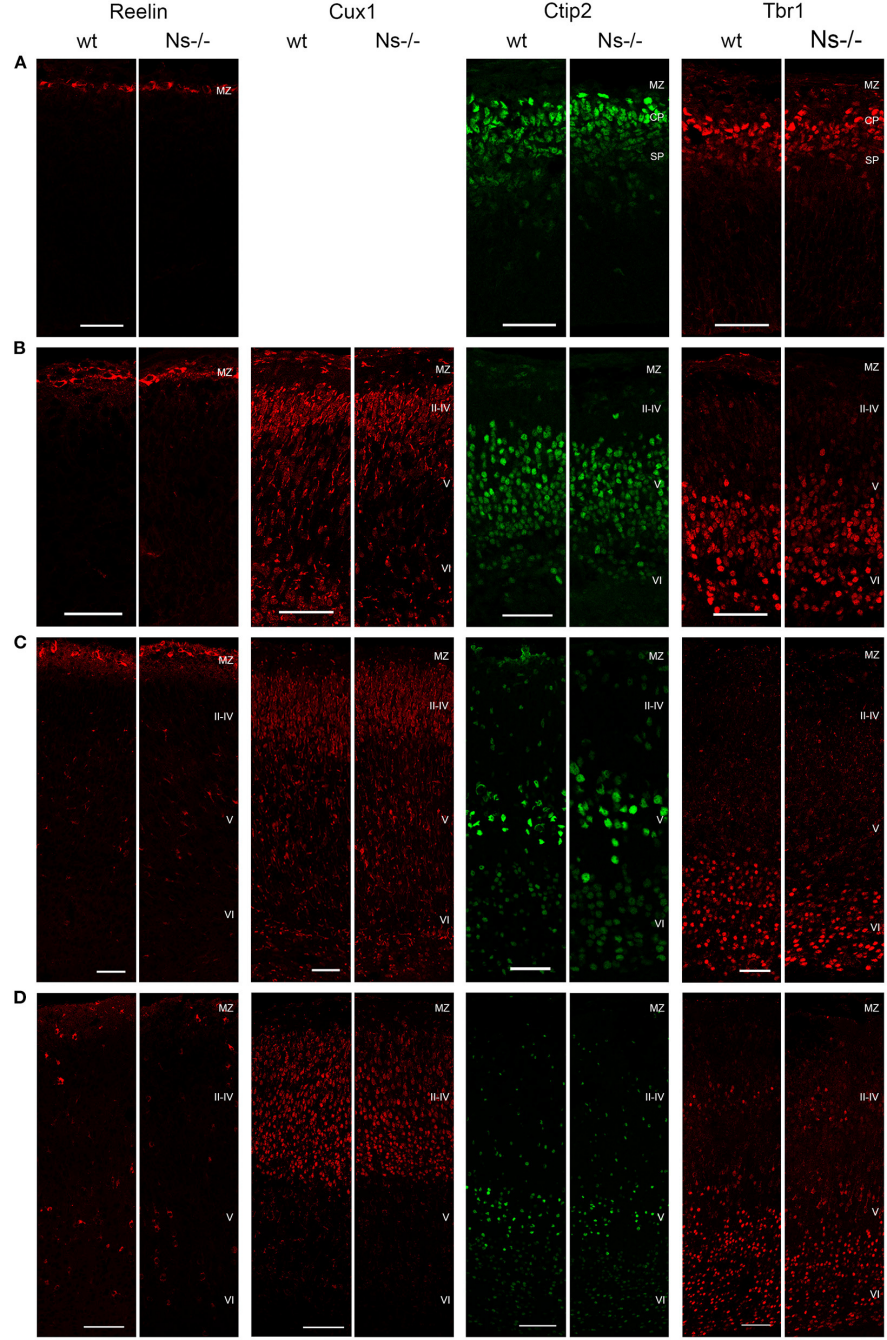
Development of the cortical layers in neuroserpin-deficient mice. In the somatosensory cortex, Reelin-positive layer I, Ctip2-positive layer V and Tbr1-positive layer VI are already formed at E13, Cux1-positive layers II-IV are generated later and can be detected starting from E16. Reelin-positive interneurons appear in the first postnatal week. Laminar organization is not altered in neuroserpin-deficient mice. Scale bars: 50 μm for E13, E16, and P0, 100 μm for 1w, *n* = 3. MZ, marginal zone; CP, cortical plate; SP, subplate.

**Figure 4:**
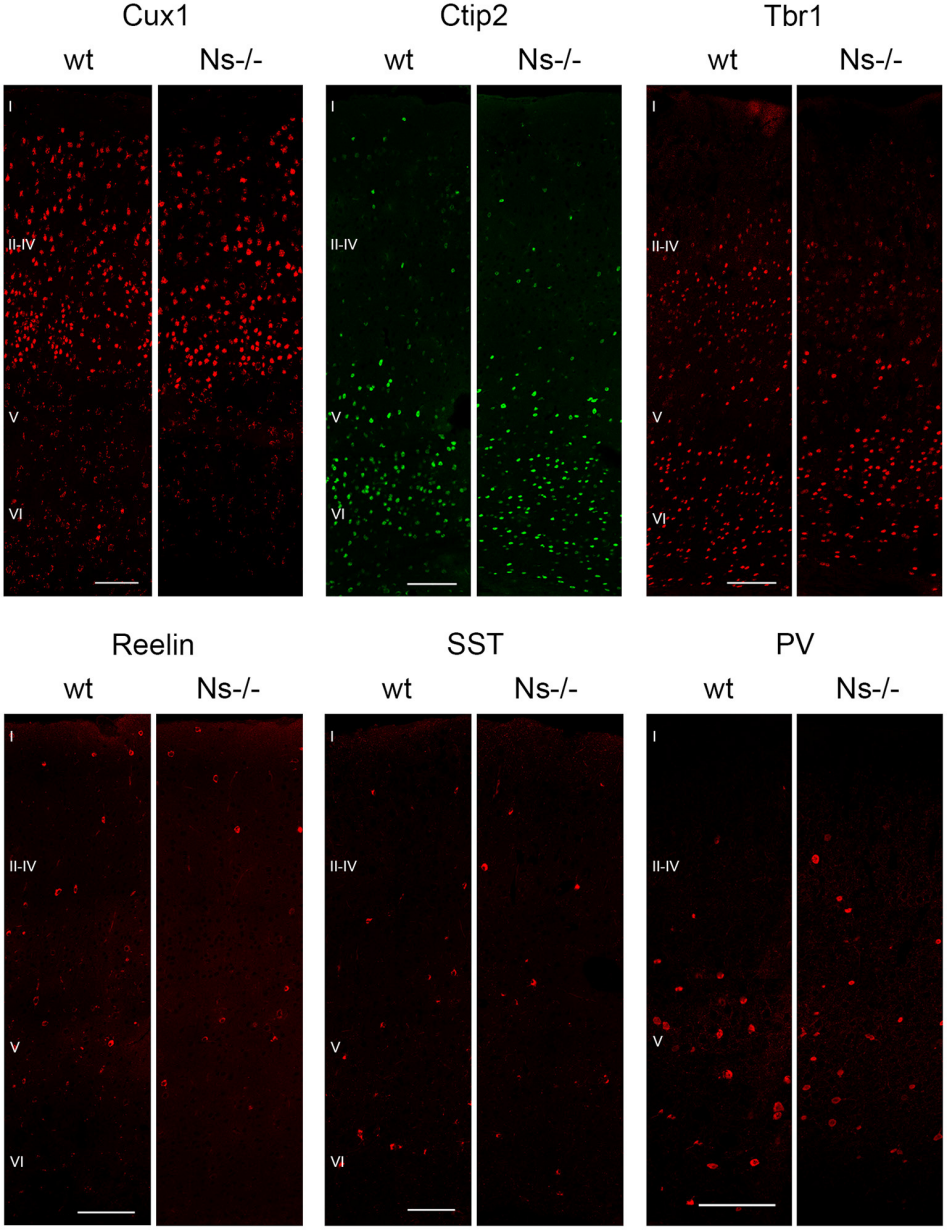
Absence of neuroserpin does not alter adult neocortical organization. Cortical layers II–IV, V and VI were visualized in the somatosensory cortex using projection neuron markers Cux1, Ctip2, and Tbr1, respectively. Distribution of GABAergic interneurons was assessed with immunostainings against Reelin, SST and PV. No obvious differences could be detected between neuroserpin-deficient mice and wild-type littermates. Scale bars: 100 μm, *n* = 3.

Because of the widespread expression of neuroserpin throughout the neocortex starting from early stages of development, and because of neuroserpin's role in formation of the hippocampus, we postulated a possible function of the serpin in corticogenesis. To investigate that, we analyzed cortex formation by comparing lamination between neuroserpin-deficient mice (Ns-/-) and wild-type littermates. Again, we concentrated on the somatosensory cortex and started our analysis at E13. At this stage, beside the marginal zone only the deep layers could be observed in the cortical plate (Figure 3A). Immunohistochemical staining with an antibody directed against Reelin allowed visualization of Cajal-Retzius cells of layer I. In both Ns-/- and wild-type groups we found an intact Reelin-positive marginal zone. Moreover, presence of Ctip2-positive and Tbr1-positive nuclei in layer V and VI, respectively, demonstrated completion of deep layer formation. No differences could be quantified in the distribution of these two classes of projection neurons between Ns-/- and wild-type counterparts (Supplementary Figure 2). In E16 brain sections, in addition to the immunostainings that we performed at E13, we also visualized Cux1-positive neurons in the newly formed upper layers II–IV (Figure 3B). Again, we did not detect any alteration in the distribution of the stained neurons throughout the neocortex between mouse groups (Supplementary Figure 2). During the first postnatal week, migration of the projection neurons within the cortex was completed. Similar staining patterns were detected in presence and absence of neuroserpin at P0 (Figure 3C), as well as at one week of age (Figure 3D) (Supplementary Figure 2). Here, the Reelin-positive staining of Cajal-Retzius cells had disappeared, stainings for Cux1, Ctip2, and Tbr1 demonstrated normal cortical lamination in wild-type as well as Ns-/- brains. In the adult mouse, besides visualization of the cortical lamination by staining Cux1, Ctip2, and Tbr1, we investigated the distribution of inhibitory GABAergic interneurons that tangentially migrate into the cortical plate (Figure 4). Similar to the situation observed in younger animals, immunoreactivity of projection neuron markers suggested intact laminar architecture in adult Ns-/- mice (Supplementary Figure 2). Furthermore, cells positive for interneuron markers Reelin, SST, and PV were detected throughout all cortical layers, with no obvious difference between mouse groups. Finally, we tested the integrity of the subplate in the somatosensory cortex of neuroserpin-deficient mice at E16 (Supplementary Figure 3). Nissl staining showed the presence of a well-developed subplate zone, and MAP2-immunostaining of cortical and subplate neurons revealed a cytoarchitectonically distinct subplate. Taken together, these data provide evidence for a normal laminar organization and GABAergic interneurons distribution in the neocortex of Ns-/- mice.

### Intact Proteolytic Processing of Reelin in Neuroserpin-Deficient Mice

**Figure 5:**
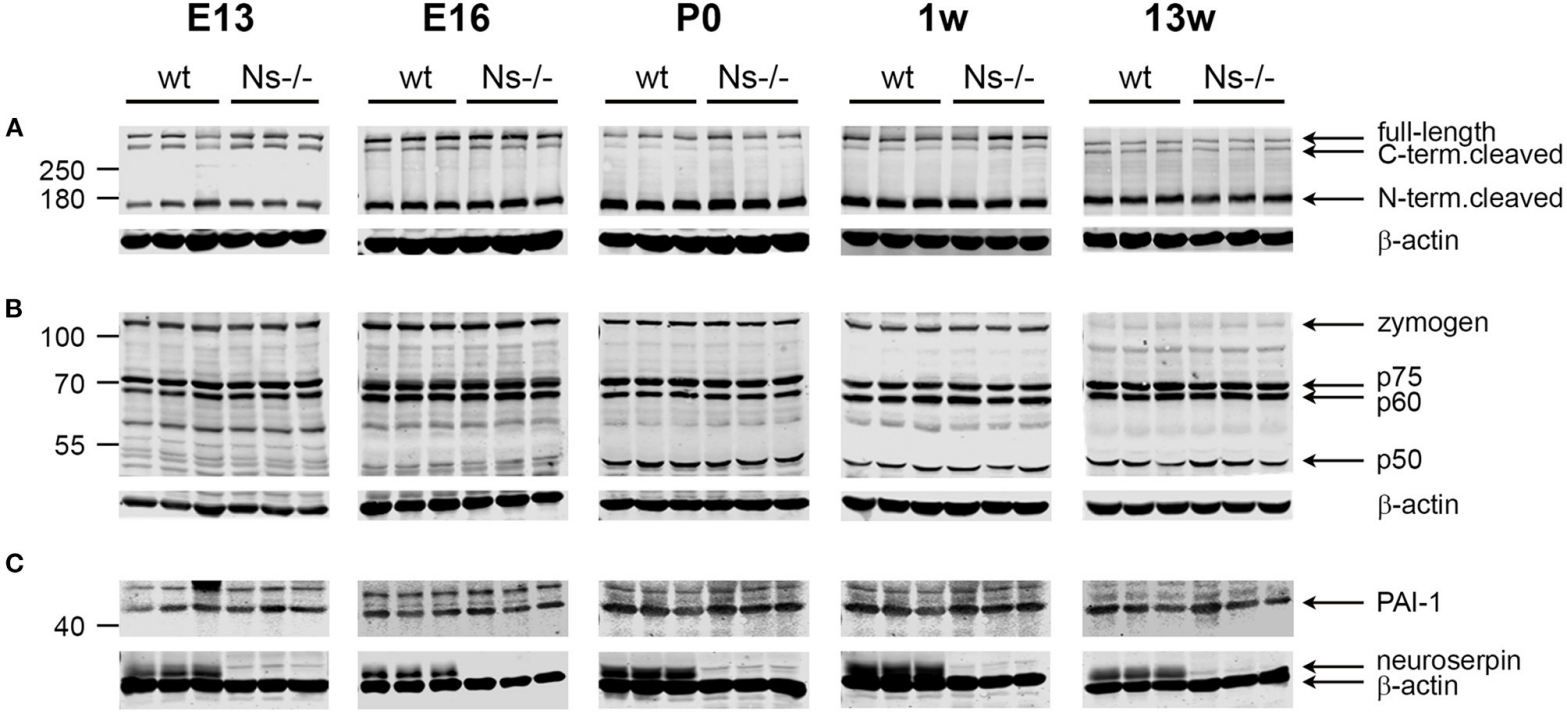
Expression and proteolytic processing of Reelin, ADAMTS-4 and PAI-1 are unaltered in the cortex of Ns-/- mice. Representative Western blots of brain extracts (E13 and E16) and neocortical extracts (P0, one and 13 weeks of age) from three different animals per group. The full-length band and the proteolytic fragments of Reelin **(A)** and the metalloprotease ADAMTS-4 **(B)**, as well as full-length PAI-1 **(C)** were visualized. Absence of neuroserpin in Ns-/- mice is demonstrated in **(C)**, where the membranes were probed with anti-neuroserpin and anti-beta actin antibodies.

Reelin is a regulator of neuronal migration and cortical lamination in the developing brain, whose function is controlled by proteolytic processing. Reelin is cleaved by two proteases: tPA, the main target of neuroserpin, and ADAMTS-4, a metalloprotease activated by tPA (Krstic et al., 2012; Lemarchant et al., 2014; Lussier et al., 2016). Since a previous study has proven an inhibitory effect of neuroserpin on tPA-dependent Reelin processing in HEK293 cells (Krstic et al., 2012), we assessed if this effect is also true *in vivo*. To this purpose, we collected brains from mice at prenatal stages (E13 and E16), and the neocortex at birth, at one week of age as well as during adulthood and compared proteolytic processing of Reelin between Ns-/- mice and wild-type littermates by Western blot (Figure 5A). Using an antibody directed against the N-Terminus of Reelin, in all five stages we detected three Reelin-positive bands: Full-length Reelin at 460 kDa, the C-terminal and the N-terminal cleaved forms at 380 and 160 kDa, respectively. Quantification of band intensity demonstrated that neither Reelin expression nor its cleavage were altered by the absence of neuroserpin at any developmental stage tested (Supplementary Figure 4). Furthermore, since tPA directly activates ADAMTS-4 (Lemarchant et al., 2014), we considered the possibility that neuroserpin could influence Reelin proteolysis by regulating tPA-dependent cleavage of the metalloprotease. In order to explore this hypothesis, we extended the Western blot analysis mentioned above to detect the different forms of ADAMTS-4, i.e., the full-length zymogen and the p75, p60, and p50 cleavage products. Whereas the band representing the full-length zymogen was particularly strong during early developmental stages and its intensity declined in the adulthood, the p50 cleavage product showed an opposite trend, with a weak band at E13 and E16 increasing in intensity in the postnatal period (Figure 5B). In contrast, production of the p60 and p75 fragments was constant over time. Importantly, we did not detect any difference in band intensity for all fragments tested between neuroserpin-deficient and wild-type cortices at any developmental stage (Supplementary Figure 4). These data demonstrate that in the absence of neuroserpin, neither cleavage of Reelin, nor activation of ADAMTS-4 are impaired. This is in line with lack of developmental abnormalities in the cortex of neuroserpin-deficient mice.

### Unaltered Levels of the Serine Protease Inhibitor PAI-1 in the Absence of Neuroserpin

PAI-1 is a serine protease inhibitor known to regulate the proteolytic function of tPA, e.g., during fibrinolysis. PAI-1 has been often discussed as a possible candidate compensating for the loss of neuroserpin in the brain of Ns-/- mice. We hypothesized that, if PAI-1 would counterbalance neuroserpin's depletion, its expression would rise in Ns-/- mouse brains. In order to test this possibility, we analyzed PAI-1 expression in the same cortical extracts mentioned above by Western blot. A band representing full-length PAI-1 was detected at 45 kDa at all developmental stages tested, in both wild-type and neuroserpin-deficient mice (Figure 5C). Importantly, quantification of band intensity did not reveal differences in PAI-1 expression between mouse groups (Supplementary Figure 4). Therefore, we conclude that absence of neuroserpin does not lead to a compensatory rise in PAI-1 synthesis.

### No Alterations have been Observed in Neuronal Anatomy and Perineuronal Net Composition in Neuroserpin-Deficient Mice

**Figure 6:**
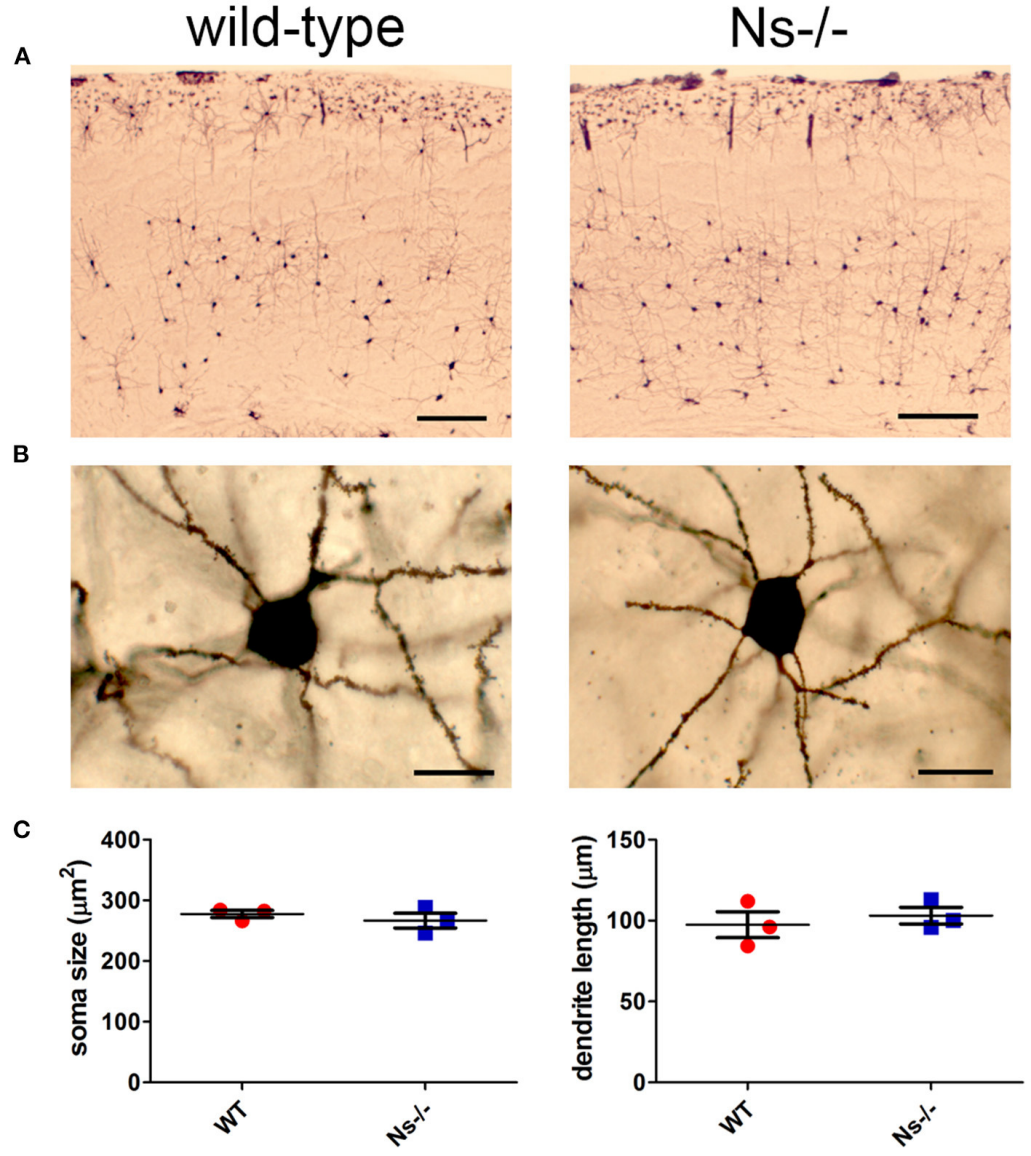
Analysis of neuronal anatomy in neuroserpin-deficient mice. **(A)** Representative Golgi-Cox stainings of the somatosensory cortex of Ns-/- mice and wild-type littermates at three weeks of age. **(B)** Pictures were taken from 30 neurons per animal, area of the soma and length of the dendrites were measured, and no significant changes were found between the two mouse groups **(C)**. Scale bars: 250 μm in **(A)**, 20 μm in **(B)**, *n* = 3.

**Figure 7:**
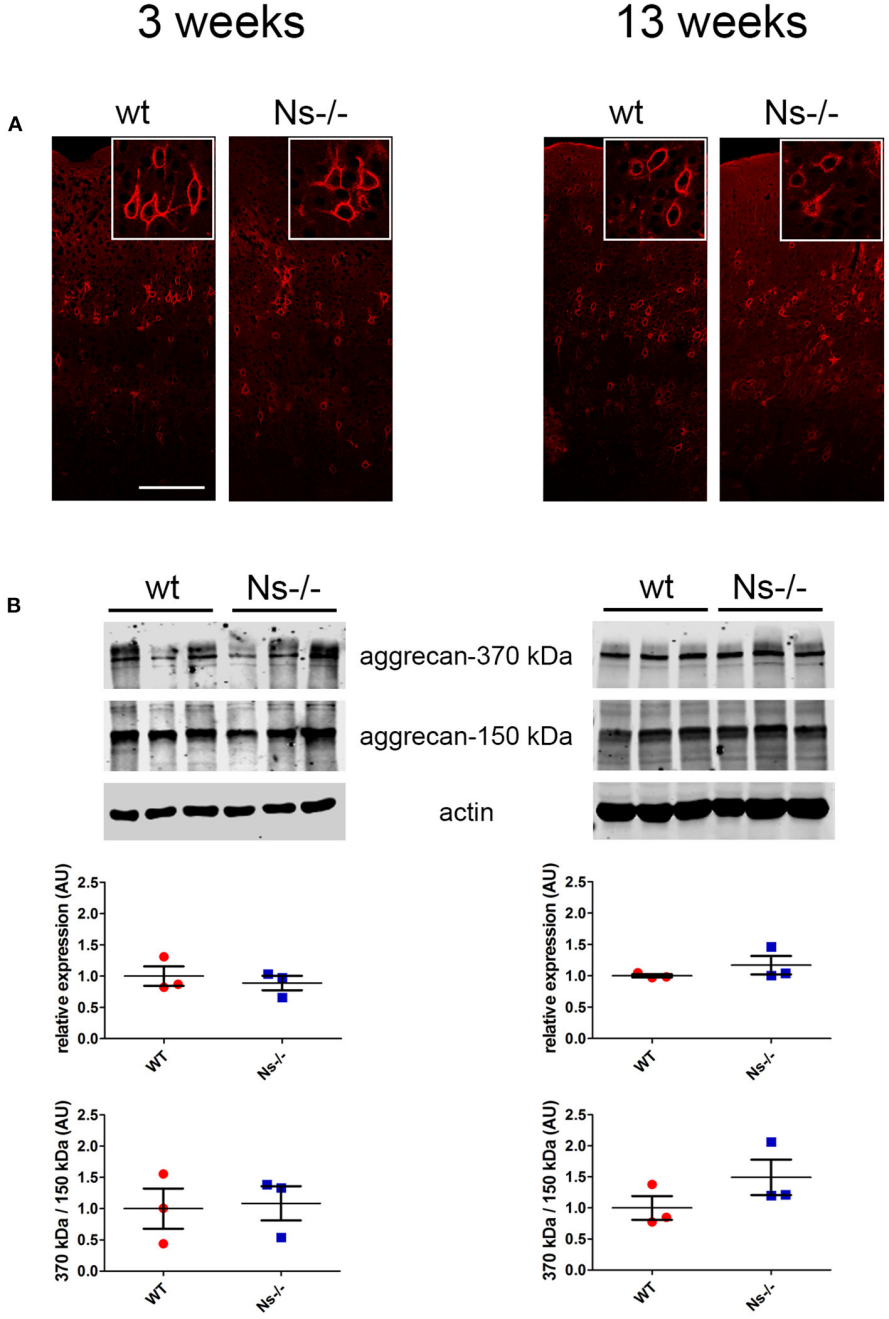
Expression and cleavage of aggrecan in Ns-/- mice. **(A)** Immunohistochemical stainings with an antibody directed against aggrecan reveal presence of the proteoglycan around distinct neurons throughout the somatosensory cortex. No obvious differences could be observed between neuroserpin-deficient mice and wild-type littermates at both three and 13 weeks of age. Scale bar: 100 μm. **(B)** Representative Western blots of neocortical extracts from three different animals per group at three and 13 weeks of age showing the 370 kDa full-length form of aggrecan and the 150 kDa proteolytic fragment. For quantification, band intensity was normalized to β-actin expression. Relative expression is presented (AU, arbitrary units), wild-type was set to 1 (mean +/– SD; three technical replicates were analyzed; *n* = 3).

Since neuroserpin is known to regulate dendritic arborization *in vitro* (Borges et al., 2010), we assessed if its absence is responsible for similar alterations in the neocortex *in vivo*. Therefore, we collected brains from wild-type and neuroserpin-deficient mice at 3 weeks of age, i.e., when neuronal migration and differentiation are completed, and the newly formed neurons have developed dendritic arbors and have assembled into circuits. We restricted our analysis to the somatosensory cortex, the same region examined in the previous experiments. Golgi-Cox impregnation allowed visualization of neurons and their dendritic branches in the deeper layers of the neocortex (Figure 6A). No gross anatomical alterations were observed between wild-type and Ns-/- mice. To further look into details of the neuronal structure, pictures were taken from 30 cells per mouse (15 per hemisphere) (Figure 6B). Surface area of the soma and average length of dendrites were measured, but no significant change was detected between the two mouse groups (Soma size: wt 277,67 +/– 10,05 μm^2^; Ns-/- 266,76 +/– 20,96 μm^2^; *p* = 0,4619. Dendrite length: wt 97,44 +/– 13,85 μm; Ns-/- 103,09 +/– 8,92 μm; *p* = 0,5848) (Figure 6C). Furthermore, we investigated aggrecan, a chondroitin sulfate proteoglycans and main components of the mature perineuronal net (PNN), specialized extracellular matrix structures found around distinct neurons and implicated in synaptic maturation (Frischknecht and Gundelfinger, 2012). Since we found altered expression and proteolytic cleavage of aggrecan in the hippocampus of juvenile neuroserpin-deficient mice, we assessed if this dysfunction is also present in the neocortex. Immunohistochemical stainings showed presence of aggrecan throughout the somatosensory cortex, especially in lower layers, strongly concentrated around the soma of certain neurons, the pattern being very similar in mice belonging to both groups (Figure 7A). Western blot analysis of cortical extracts revealed both full-length aggrecan and 150 kDa band representing the main proteolytic fragment, but no difference in expression and cleavage could be detected between neuroserpin-deficient and control mice at three as well as at 13 weeks of age (Figure 7B).

### Analysis of Neocortical Synapses in Adult Ns-/- Mice

**Figure 8:**
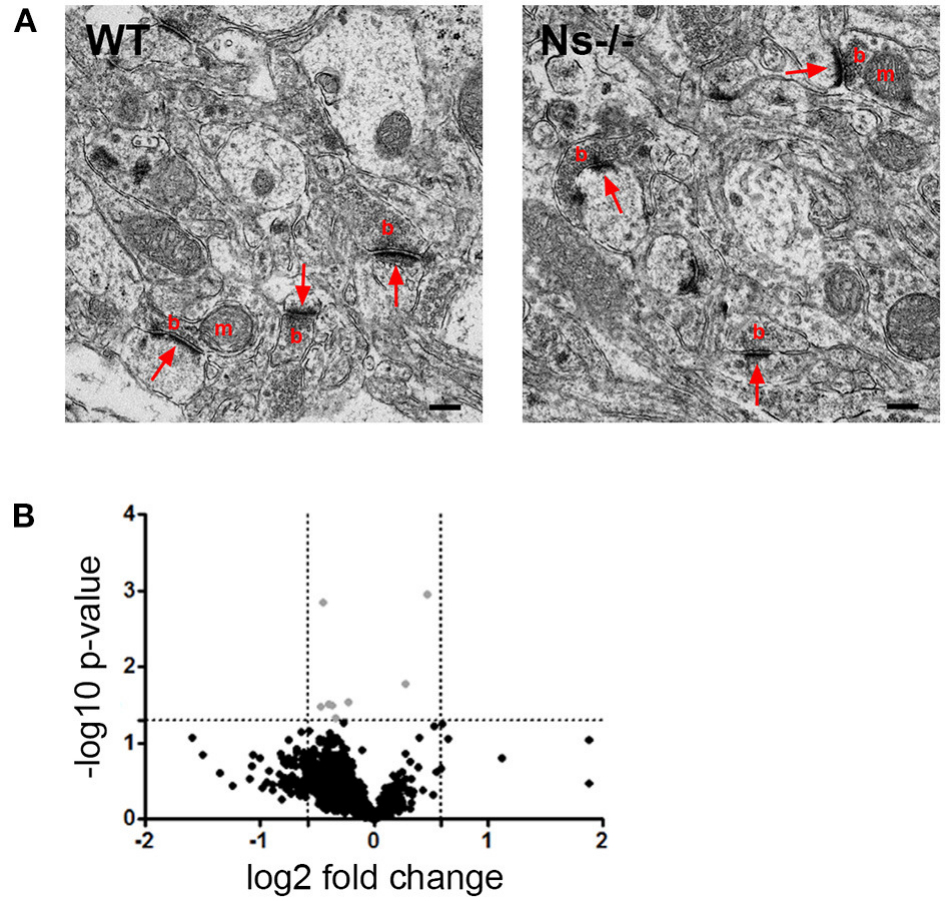
No differences were detected in neocortical synapses of adult Ns-/- mice. **(A)** Electron micrographs of synapses in the somatosensory cortex of control (WT) and neuroserpin-deficient (Ns-/-) mice. No alterations in synaptic morphology was observed in the absence of neuroserpin. Shown are synaptic contacts consisting of presynaptic boutons (b) occasionally containing mitochondria (m) and postsynaptic densities (arrows). Scale bars: 250 nm, *n* = 3. **(B)** Volcano plot illustrating *t*-test significantly (*p*-value < 0.05) differential abundant proteins between neocortical synaptosomes isolated from neuroserpin-deficient mice and wt littermates. The -log10 *p*-value is plotted against the log2-fold change. The non-axial, horizontal line represents the significance threshold (*p*-value < 0.05). Vertical non-axial lines represent the log2 value of the +/– 1.5-fold change cutoff. No protein exceeded the *p*-value < 0.05 as well as the 1.5-fold change cutoff among the compared groups. Black dots, *p*-value > 0.05; gray dots, *p*-value < 0.05 and fold change < 1.5, *n* = 4.

Since absence of neuroserpin alters synaptic morphology and plasticity in the adult mouse hippocampus (Reumann et al., 2017), we hypothesized that, despite normal cortical development, in the neocortex as well neuroserpin could exert a regulatory function at synaptic level in the adult. In order to investigate this, we performed ultrastructural analysis of synapses of the primary somatosensory cortex in adult neuroserpin-deficient mice and wild-type littermates (Figure 8A). Electron micrographs revealed a heterogenic population of regularly formed synapses in both mouse groups. Synaptic contacts showed typical asymmetric postsynaptic densities and presynaptic boutons. Many densely packed vesicles, and occasionally mitochondria, were detected in the presynaptic terminals. All in all, no obvious difference in the overall morphology of synaptic contacts was observed. Furthermore, although cortical synaptic morphology is preserved in the absence of neuroserpin, we considered the possibility that neuroserpin deficiency could lead to synaptic dysfunction at protein level. To evaluate this, we isolated synaptosomes from the neocortex of adult neuroserpin-deficient mice and control littermates and subjected them to differential quantitative proteomics to compare the synaptic proteome between both mouse groups. A total number of 1,715 proteins were identified, of which 1,268 were found in at least three out of four animals of both wt and Ns-/- group (Supplementary Table 2). Brain and synaptic proteins, as well as extracellular matrix components and proteins involved in vesicular trafficking processes, were found to be highly represented among the identified proteins (Supplementary Table 2). Principle component analysis as well as unsupervised hierarchical clustering revealed no clear distinguishability between Ns-/—and wt mice at the proteomic level (Figure 8B). T-testing revealed only eight proteins significantly differentially abundant between both compared groups at a *p*-value < 0.05. However, no protein exceeded a 1.5-fold change difference cutoff between the queried groups, indicating that neuroserpin deficiency does not significantly alter the composition of the synaptic proteome in the neocortex region.

## Discussion

In recent years, investigations have examined the role of neuroserpin in the hippocampus and demonstrated its indispensable function in the proper formation of this brain region (Hermann et al., 2020). In the adulthood, lack of neuroserpin is responsible for altered spine-synapse density and synaptic plasticity in the hippocampus. Consistent with this, neuroserpin-deficient mice show cognitive and sociability deficits in hippocampal-dependent behavioral tasks (Reumann et al., 2017). In view of its key role in the hippocampus, we extended the analysis of neuroserpin function to the neocortex, a region presenting strong expression of the serpin and critical for encoding and retrieval of memories.

First, we performed an accurate investigation of neuroserpin expression in the neocortex. We observed a broad expression at low levels during development being distributed more differentially in the mature tissue. This expression pattern has already been described for the hippocampus (Hermann et al., 2020) and is in line with the observation made by Krueger and colleagues using *in situ* hybridization according to which neurons migrating in the cortical plate express neuroserpin weakly, but once they have settled in the cortical plate and have differentiated they contain larger amounts of neuroserpin mRNA (Krueger et al., 1997). Based on this observation we hypothesized that, in contrast to a ubiquitous role throughout the tissue during development, in the adult cortex neuroserpin could be specifically involved in regulating proteolytic processes in a particular subset of cells, therefore elucidating the nature of cells producing neuroserpin could provide further insight into its function. To find out which cells express neuroserpin, we analyzed colocalization with different neuronal and glial markers by immunohistochemistry. Similar to the situation in the hippocampus (Hermann et al., 2020), we did not detect expression of neuroserpin in GFAP-positive astrocytes, Iba1-positive microglia and Olig2-positive oligodendrocytes, suggesting a neuronal origin. Still, since investigated markers do not represent the entirety of glial cells, we cannot exclude neuroserpin expression by further non-neuronal subpopulations. We found neuroserpin immunoreactivity in excitatory projection neurons of upper (Cux1-positive) and deep (Tbr1-positive) layers during development, but in the mature cortex colocalization with Tbr1 was restricted to few cells located at the bottom of layer VI. Although a colocalization study would be necessary to draw conclusions, we postulate that these Tbr1-neuroserpin-double positive cells could reside in the subplate, a region known to express high levels of the serpin during cortical development (Kondo et al., 2015). Moreover, neuroserpin reactivity was observed in PV-, SST-, and Reelin-positive GABA-ergic interneurons throughout the cerebral cortex. This broad expression of neuroserpin in both inhibitory interneurons and excitatory pyramidal neurons is in line with the observations previously described in human tissue by Adorjan et al. (2019), but unfortunately it does not allow to draw any conclusion about a possible role for neuroserpin in the cerebral mouse cortex. Still, since our investigation included only few markers for projection neurons and interneurons, we cannot exclude that further examinations will reveal specific neuroserpin functions linked to non-tested neuronal subtypes.

Next, we assessed a possible colocalization of neuroserpin and its target protease, tPA, in the somatosensory cortex at P0, the developmental stage at which the expression of neuroserpin reaches its highest level (Krueger et al., 1997). We found tPA-expressing cells throughout the cortex, in line with previous works describing presence of the serine protease in layers II–VI in glutamatergic pyramidal neurons, as well as endothelial cells and some glial cells (Louessard et al., 2016; Stevenson and Lawrence, 2018). Although co-expression of neuroserpin and tPA in the human central nervous system, in particular in regions with neuronal plasticity and motor learning, has previously been reported (Teesalu et al., 2004), the possibility that both proteins are produced by the same cell has never been investigated. We show here that, although a strong immunoreactivity for neuroserpin and its target is observed in the somatosensory cortex, we did not detect cells producing both proteins. This does not exclude that the proteins, after being secreted from neighboring cells, interact in the extracellular environment, where neuroserpin could regulate the proteolytic activity of tPA.

Having observed the presence of neuroserpin in the neocortex starting from early developmental stages, and knowing its key role in hippocampal development, we asked if its absence would influence corticogenesis. Therefore, we analyzed formation of the cortex histologically by staining markers of pyramidal neurons located in the different layers and of inhibitory interneuron in neuroserpin-deficient mice and wild-type littermates, and we did not detect any difference between mouse groups. Moreover, since the subplate exerts a key function during corticogenesis, e.g., in controlling migration of cortical neurons (Wang et al., 2010) we tested its integrity, but we failed to observe obvious changes in its cytoarchitcture. Though a more in-depth analysis will be necessary to exclude defects in the functionality of the subplate in the absence of neuroserpin, the data presented here argue for a normal lamination of the neocortex in neuroserpin-deficient mice. Interestingly, lack of tPA, the main target of neuroserpin, results in deficits in neuronal migration and positioning at very early developmental stages, thereby highlighting the essential role of this serine protease in cortical formation (Pasquet et al., 2019). However, the same phenotype is observed in the presence of a mutant form of tPA that cannot bind NMDAR, suggesting that NMDAR binding, more than the proteolytic function of tPA, is necessary to control neuronal migration during corticogenesis. If this is true, neuroserpin function as tPA inhibitor would be dispensable in this context, a finding which is in line with data presented here. Though, we cannot exclude that during cortical development neuroserpin could inhibit serine proteases other than tPA or act independently from its function as a serpin. An alternative non-inhibitory mechanism through which neuroserpin may act could be by binding to a transmembrane receptor. Neuroserpin is known to bind to and be internalized by the low-density lipoprotein receptor-related protein (LRP) (Makarova et al., 2003), a protein that plays a role during brain development and in synaptic functions (Narita et al., 1997; May et al., 2004). Moreover, lack of lamination defects in neuroserpin-deficient mice could be due to compensatory effects by other proteins. In this respect we investigated the expression of PAI-1, another regulator of tPA activity. Lack of an overexpression of PAI-1 in the neocortex of neuroserpin-deficient mice speaks against a compensatory inhibition by PAI-1. Still, further investigations are needed to study the compensatory effect more closely, since it could be detectable only by a conditional knockout of neuroserpin at a later developmental stages.

Reelin is a glycoprotein expressed and secreted in many organs (Forster et al., 2010). Interestingly, only the brain is affected by mutations leading to loss of function of the protein (D'arcangelo et al., 1995). The most striking phenotype is a defect in lamination of cerebellum and neocortex due to incorrect neuronal migration. In the cerebral cortex, Reelin is cleaved by different proteases at two specific sites, leading to the production of three major fragments that, together with the full-length variant, have been observed in the developing as well as in the adult brain (Trotter et al., 2014). Recently, a study by Krstic et al. demonstrated that tPA cleaves Reelin at the C-terminus *in vitro* and in HEK293 cells and this proteolytic step can be inhibited by co-expressing neuroserpin (Krstic et al., 2012). We tested this *in vivo* in mouse cortical extracts, but we could not detect changes in Reelin expression or cleavage between neuroserpin-deficient mice and wild-type littermates. Moreover, in the same study Krstic and colleagues proved cleavage of Reelin at both N- and C-terminus by ADAMTS-4, a metalloprotease activated by tPA (Lemarchant et al., 2014). Although this finding could not be confirmed in cultured cerebral cortical neurons (Hisanaga et al., 2012), it offers another regulatory mechanism how neuroserpin could control Reelin cleavage. Again, we checked this by analyzing ADAMTS-4 by Western blot in cortical extracts from neuroserpin-deficient mice. We did not detect any alteration in the abundance of full-length and proteolytic fragments of ADAMTS-4, suggesting that either ADAMTS-4 cleavage by tPA is not taking place in the cortex under physiological conditions, or another serpin regulates this process, thus compensating for neuroserpin absence in knockout mice. Taken together, unaltered Reelin expression and cleavage in the cortex of mice deficient in neuroserpin is in agreement with absence of deficits in corticogenesis detected in this work. Our data contradict the inhibitory effect of neuroserpin on Reelin cleavage observed in HEK293 cells. It is conceivable that this could be relevant in other brain regions, e.g., in the adult hippocampus in the context of synaptic plasticity, a process regulated by neuroserpin. Moreover, it could be hypothesized that neuroserpin function on Reelin cannot be observed under basal conditions but only after synaptic potentiation. A similar situation has been described in mice lacking tPA, where upregulation of tPA-dependent Reelin processing is observed only after induction of NMDAR-independent long-term potentiation with a potassium channel blocker (Trotter et al., 2014).

Although neuroserpin-deficient mice do not show deficits in cortical layer formation, we assessed the possibility that, similarly to the situation in the hippocampus, neuroserpin could control neuronal maturation and synaptic phenotype in the neocortex as well. Therefore, we concentrated our analysis on postnatal development. We examined average length of dendrites and soma size in mice at this stage by staining brains with the Golgi-Cox method and quantifying these parameters morphologically, but we did not find any difference between neuroserpin-deficient and control mice. Based on observations from an *in vitro* study with cultured hippocampal neurons, where increased dendritic protrusion has been detected in neuroserpin-overexpressing cells (Borges et al., 2010), we would have expected a dendritic phenotype in mice lacking the serpin. So again, this either speaks for a hippocampal-specific effect, or it may be caused by protein overexpression. Another reason for the lack of a dendritic phenotype could be due to the limitations of our analysis. The Golgi-Cox protocol impregnates randomly neurons, and different cell types have different propensity to stain (Pasternak and Woolsey, 1975; Shimono and Tsuji, 1987). Moreover, neurons in the upper layers are spared from the staining, and our analysis was restricted to the somatosensory cortex, therefore our results may not be representative and thus do not allow a general statement on the dendritic phenotype in neuroserpin-deficient mice. Furthermore, we did not detect any difference in expression and proteolytic cleavage of aggrecan in the neocortex at three weeks of age. Aggrecan is the main component of PNN, extracellular matrix structures of the central nervous system typically localized around some parvalbumin-positive, GABAergic inhibitory interneurons in the cerebral cortex. Aggrecan expression gradually increases postnatally and correlates with the process of synaptic maturation and stabilization observed at the end of the critical period (Frischknecht and Gundelfinger, 2012). We previously reported that neuroserpin influences aggrecan expression and cleavage at three weeks of age in the hippocampus (Hermann et al., 2020), however, again, this role does not seem to be exerted in the cerebral cortex.

Lastly, we analyzed cortical synapses in adult mice by a quantitative proteomic analysis of synaptosomes, structures consisting of a pre-synaptic terminal containing synaptic vesicles, dense-core vesicles and mitochondria, and a post-synaptic density attached to the pre-synaptic active zone (Phelan and Gordon-Weeks, 1997). This analysis allowed to quantify 1,268 proteins in at least three out of four tested animals for every group. The dominant presence of many brain- and synapse-specific proteins, indicated by the statistically significant enrichment of brain and synapse specific gensets, argues for successful sample preparation. However, the composition of the synaptic proteome was not significantly altered by the loss of neuroserpin. Together with normal morphology of synaptic contacts observed at electron microscopic levels and with unaltered expression and processing of aggrecan in the adult cortex, these data speak against a role of neuroserpin in regulation of cortical synaptic composition. However, we cannot exclude that subtler changes could be present. A more in-depth analysis of the synapses involving 3D-reconstruction may reveal minor morphological alterations. Moreover, analysis of the proteome of the neocortex without synaptic enrichment *via* synaptosome isolation could disclose mechanisms of action that take place beyond the synaptic compartment.

## Conclusions

The present work shows that neuroserpin is strongly expressed in the neocortex starting from early developmental stages until adulthood. We observed expression in Cux1- and Tbr1-positive excitatory glutamatergic projection neurons and PV-, SST- and, Reelin-positive inhibitory GABA-ergic interneurons but not in glial cells. During formation of the neocortex, neuroserpin does not contribute to cortical lamination and does not regulate cleavage of Reelin. Furthermore, under physiological conditions, with all limitations of our methods, we did not detect alterations in neuronal maturation and synaptic protein composition. This does not exclude that a phenotype could be revealed under stressful conditions, like ischemia or a viral infection, and may be responsible for neurological dysfunction later in life. Therefore, further work is needed to explore the presence of subtle, yet undetected alterations in the neocortex of neuroserpin-deficient mice.

## Data Availability Statement

The datasets presented in this study can be found in online repository. The name of the repository and accession number can be found in the article.

## Ethics Statement

The animal study was reviewed and approved by Behörde für Lebensmittelsicherheit und Veterinärwesen, Hamburg, Germany (registration number ORG-739) and ethical committee CENOMEXA, Caen, France (identification number #10638).

## Author Contributions

GG and MG designed the study, GG conceived and planned the experiments, DK, RR, KS, HV, SD, MS, and GG carried out the experiment. MD analyzed the experimental data. GG supervised the project. GG and DK wrote the paper with input from all authors. All authors contributed to the interpretation of the results.

## Conflict of Interest

The authors declare that the research was conducted in the absence of any commercial or financial relationships that could be construed as a potential conflict of interest.

## Supplementary Material

Supplementary Figure 1 Colocalization of neuroserpin and its target protease tPA. Pregnant mice were in utero electroporated at E14 with the pPlat-GFP plasmid, resulting in detection of the GFP in cells expressing tPA. Co-electroporation with the pCAG-RFP plasmid allowed localization of the electroporated area (RFP staining). Mice were killed at P0, brains were fixed and sections of the somatosensory cortex stained with an anti-neuroserpin antibody. Visualization of tPA-positive cells was enhanced by staining with an anti-GFP antibody. Both neuroserpin (Ns) and tPA are expressed throughout the cerebral cortex, however cells expressing both proteins cannot be observed. White box represents the area shown at higher magnification in the top row. Scale bar: 50 μm.

Supplementary Figure 2 Quantification of cortical immunofluorescence signal. Cortices were divided into 10 equally sized bins, the percentage of positive immunofluorescence to bin area was calculated for the cortical markers Cux1, Ctip2, and Tbr1 at E13, E16, P0, one and 13 weeks of age. Values of wt mice (red bars) were compared to Ns-/- (blue bars), no significant difference was observed at any stage. *n* = 3 animals, bars indicate mean, error bars indicate SD.

Supplementary Figure 3 Analysis of the subplate in Ns-/- mice at E16. **(A)** Nissl staining of the developing cortex of wild-type and Ns-/- mice at E16. **(B)** The same region was stained with an antibody directed against the neuronal marker MAP2 (green). Nuclei were counterstained with DAPI (blue). CP, cortical plate; SP, subplate. Scale bar: 50 μm.

Supplementary Figure 4 Quantification of Reelin, ADAMTS-4, and PAI-1 expression and cleavage. Intensity of each band was normalized to β-actin expression. Relative expression is presented (AU, arbitrary units), expression for the wt group was set to 1. *n* = 3 animals; three technical replicates were analyzed; bars indicate mean, error bars indicate SD.

Supplementary Table 1 Animals used in this study. Number and age of the mice included in this study are listed, as well as brain area and analysis that has been performed with the tissues.

Supplementary Table 2 List of proteins identified by differential quantitative proteomics. Synaptosomes were isolated from the neocortex of adult neuroserpin-deficient mice and control littermates and analyzed by differential quantitative proteomics to assess synaptic alterations between both mouse groups (*n* = 4). In red are proteins with *p*-value < 0.05.

## Abbreviations

AU: arbitrary unit
CP: cortical plate
E: embryonic day
GFAP: glial fibrillary acidic protein
GFP: green fluorescence protein
Ns: neuroserpin
Ns-/-: neuroserpin-deficient mice
P: postnatal day
PAI-1: plasminogen activator inhibitor-1
PNN: perineuronal net
PV: parvalbumin
RFP: red fluorescence protein
RT: room temperature
SP: subplate
SST: somatostatin
tPA: tissue-type plasminogen activator
wt: wild-type.
