# Supplementary figures and images for: Neuroserpin Is Strongly Expressed in the Developing and Adult Mouse Neocortex but Its Absence Does Not Perturb Cortical Lamination and Synaptic Proteome
Source: Front Neuroanat. 2021 Feb 23;15:627896. doi: 10.3389/fnana.2021.627896 (PMC7940840; doi:10.3389/fnana.2021.627896)

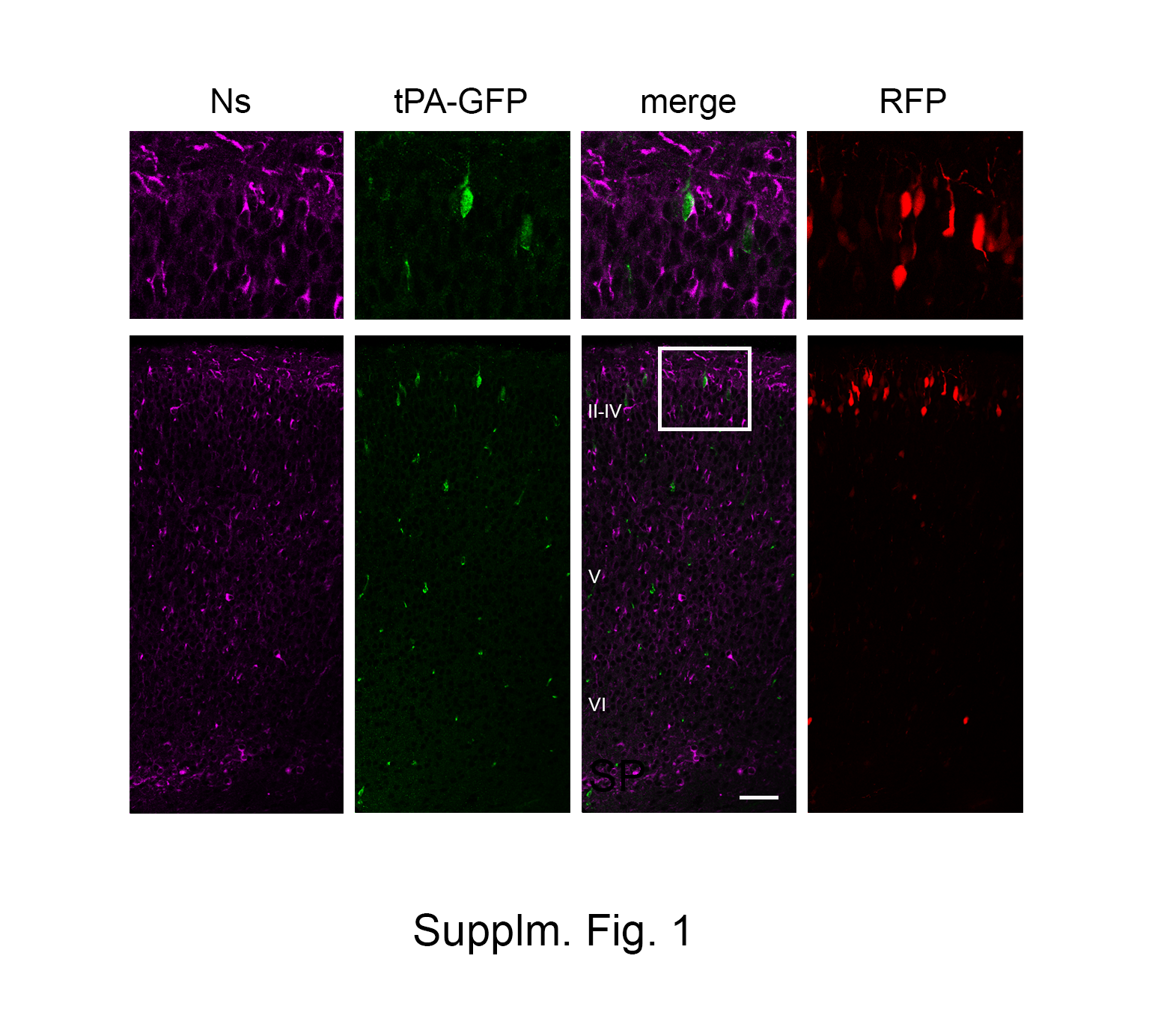

Supplement: Supplementary Figure 1 — Colocalization of neuroserpin and its target protease tPA. Pregnant mice were in utero electroporated at E14 with the pPlat-GFP plasmid, resulting in detection of the GFP in cells expressing tPA. Co-electroporation with the pCAG-RFP plasmid allowed localization of the electroporated area (RFP staining). Mice were killed at P0, brains were fixed and sections of the somatosensory cortex stained with an anti-neuroserpin antibody. Visualization of tPA-positive cells was enhanced by staining with an anti-GFP antibody. Both neuroserpin (Ns) and tPA are expressed throughout the cerebral cortex, however cells expressing both proteins cannot be observed. White box represents the area shown at higher magnification in the top row. Scale bar: 50 μm. [file Image_1.TIF]

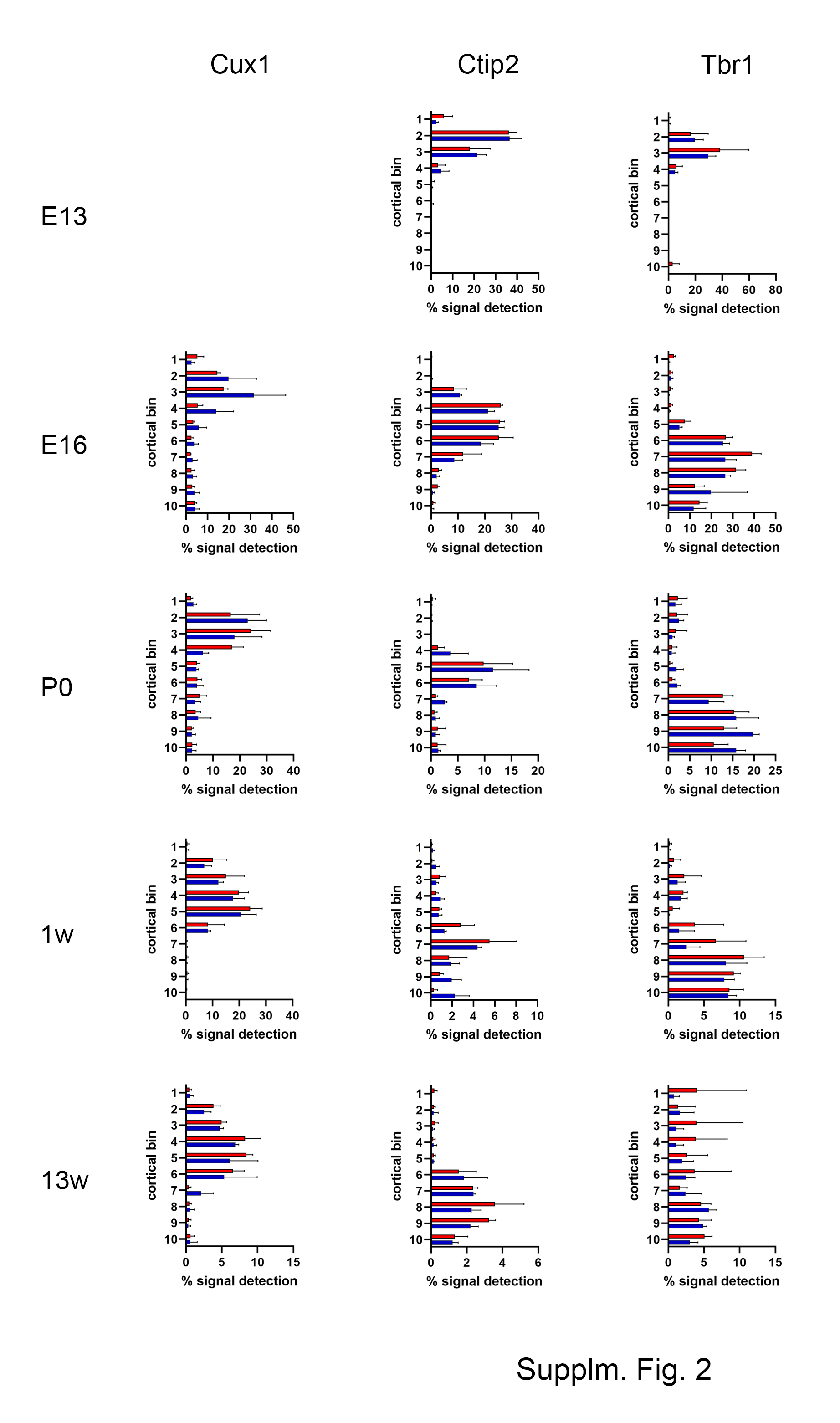

Supplement: Supplementary Figure 2 — Quantification of cortical immunofluorescence signal. Cortices were divided into 10 equally sized bins, the percentage of positive immunofluorescence to bin area was calculated for the cortical markers Cux1, Ctip2, and Tbr1 at E13, E16, P0, one and 13 weeks of age. Values of wt mice (red bars) were compared to Ns-/- (blue bars), no significant difference was observed at any stage. n = 3 animals, bars indicate mean, error bars indicate SD. [file Image_2.TIF]

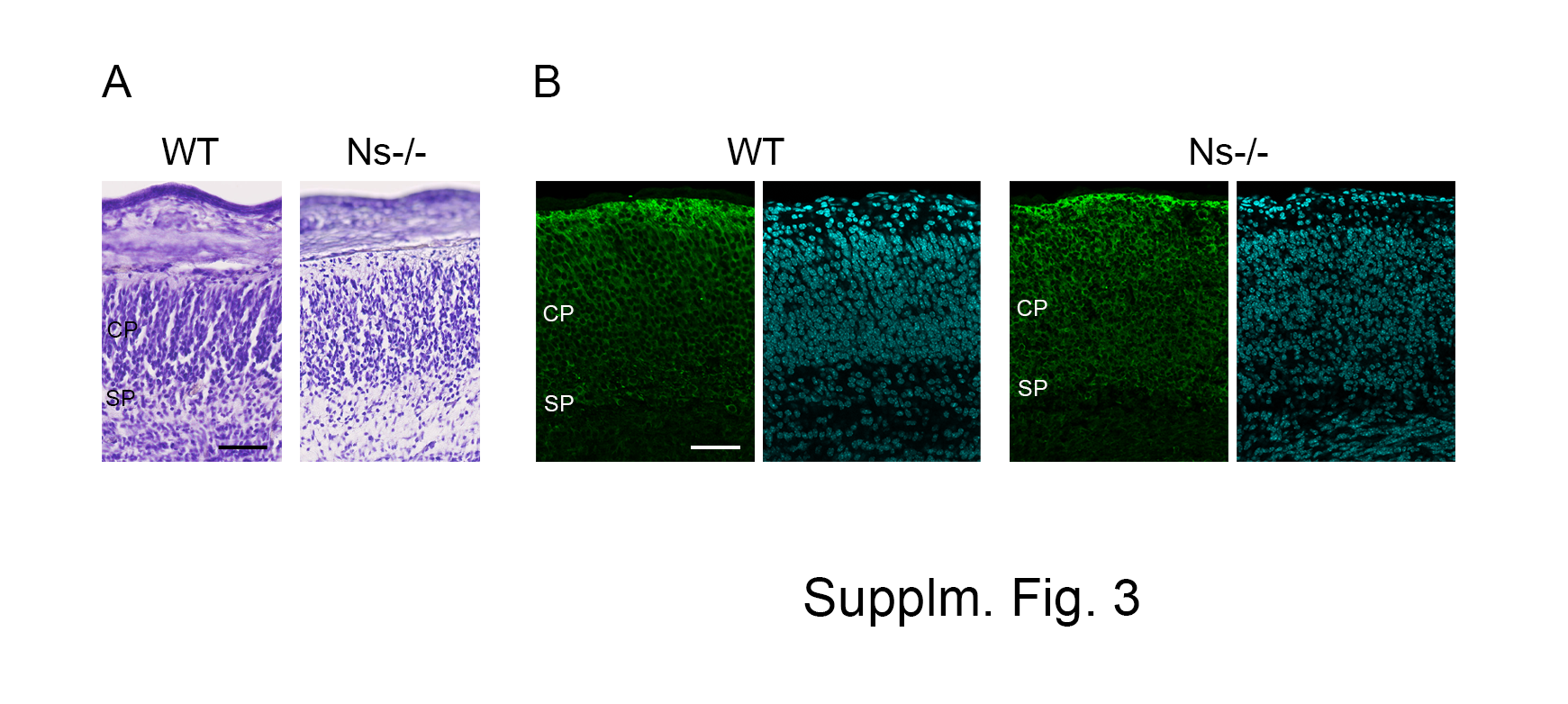

Supplement: Supplementary Figure 3 — Analysis of the subplate in Ns-/- mice at E16. (A) Nissl staining of the developing cortex of wild-type and Ns-/- mice at E16. (B) The same region was stained with an antibody directed against the neuronal marker MAP2 (green). Nuclei were counterstained with DAPI (blue). CP, cortical plate; SP, subplate. Scale bar: 50 μm. [file Image_3.TIF]

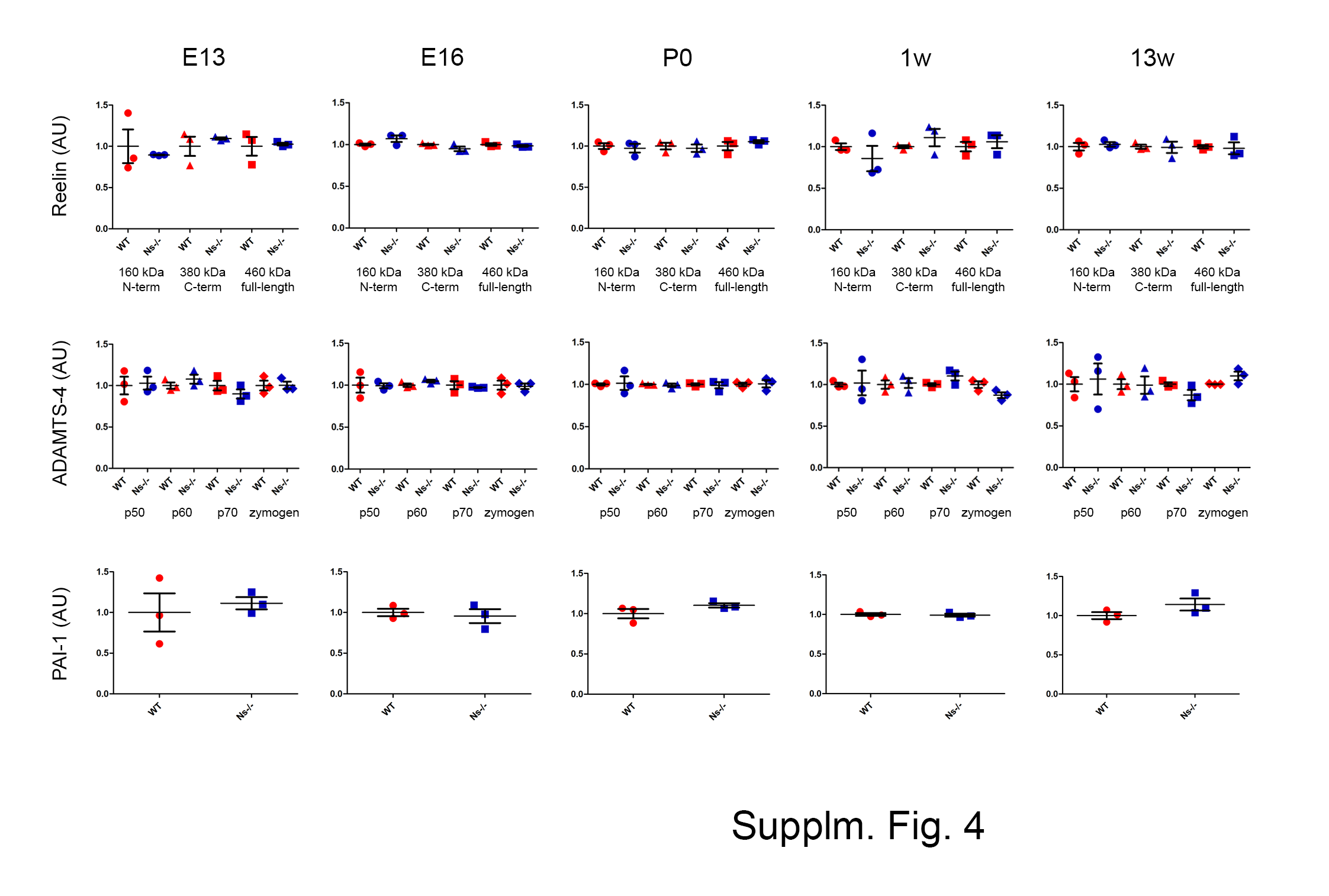

Supplement: Supplementary Figure 4 — Quantification of Reelin, ADAMTS-4, and PAI-1 expression and cleavage. Intensity of each band was normalized to β-actin expression. Relative expression is presented (AU, arbitrary units), expression for the wt group was set to 1. n = 3 animals; three technical replicates were analyzed; bars indicate mean, error bars indicate SD. [file Image_4.TIF]
